# The HIV Modes of Transmission model: a systematic review of its findings and adherence to guidelines

**DOI:** 10.7448/IAS.17.1.18928

**Published:** 2014-06-23

**Authors:** Zara Shubber, Sharmistha Mishra, Juan F. Vesga, Marie-Claude Boily

**Affiliations:** 1 Department of Infectious Disease Epidemiology, Imperial College London, London, UK; 2 St. Michael’s Hospital, University of Toronto, Toronto, Canada

**Keywords:** HIV, HIV infection, HIV prevention policy, Modes of Transmission model, epidemic appraisal, key populations

## Abstract

**Introduction:**

The HIV Modes of Transmission (MOT) model estimates the annual fraction of new HIV infections (FNI) acquired by different risk groups. It was designed to guide country-specific HIV prevention policies. To determine if the MOT produced context-specific recommendations, we analyzed MOT results by region and epidemic type, and explored the factors (e.g. data used to estimate parameter inputs, adherence to guidelines) influencing the differences.

**Methods:**

We systematically searched MEDLINE, EMBASE and UNAIDS reports, and contacted UNAIDS country directors for published MOT results from MOT inception (2003) to 25 September 2012.

**Results:**

We retrieved four journal articles and 20 UNAIDS reports covering 29 countries. In 13 countries, the largest FNI (range 26 to 63%) was acquired by the low-risk group and increased with low-risk population size. The FNI among female sex workers (FSWs) remained low (median 1.3%, range 0.04 to 14.4%), with little variability by region and epidemic type despite variability in sexual behaviour. In India and Thailand, where FSWs play an important role in transmission, the FNI among FSWs was 2 and 4%, respectively. In contrast, the FNI among men who have sex with men (MSM) varied across regions (range 0.1 to 89%) and increased with MSM population size. The FNI among people who inject drugs (PWID, range 0 to 82%) was largest in early-phase epidemics with low overall HIV prevalence. Most MOT studies were conducted and reported as per guidelines but data quality remains an issue.

**Conclusions:**

Although countries are generally performing the MOT as per guidelines, there is little variation in the FNI (except among MSM and PWID) by region and epidemic type. Homogeneity in MOT FNI for FSWs, clients and low-risk groups may limit the utility of MOT for guiding country-specific interventions in heterosexual HIV epidemics.

## Introduction

In 2002, the HIV Modes of Transmission (MOT) model was developed to help inform and focus country-specific HIV prevention policies [1, 2]. The MOT – a simple, static, mathematical model – divides the adult population into the following mutually exclusive risk groups: female sex workers (FSW); clients; men who have sex with men (MSM); people who inject drugs (PWID); individuals with casual partners; those at lower risk (i.e. in monogamous partnerships); and the partners of these different risk groups [3]. Parameterization of the model requires data on population sizes, HIV and sexually transmitted infection (STI) prevalence, and sexual behaviour of each risk group to estimate the HIV incidence, and the annual fraction of new HIV infections (FNI) acquired by each risk group. The FNI, which is the main outcome derived with the MOT, is the estimated fraction of all new HIV infections among adults that is acquired by one specific risk group in one year. The MOT results are usually used as part of the wider UNAIDS’ “Know your epidemic, Know your response” synthesis to help allocate HIV prevention resources to the most afflicted risk groups. Before the MOT, the numerical proxy method was often used to help allocate prevention resources. The numerical proxy classifies epidemics as “low-level” or “concentrated” if HIV prevalence remains below 1% in the general population, and remains below or exceeds 5% in a high-risk group, respectively. An epidemic is “generalized” if HIV prevalence among the general population exceeds 1%. With this framework, it is recommended to focus on high-risk groups in concentrated and low-level epidemics, and to target “all segments of society” in generalized epidemics [4, 5].

In 2002, the HIV Modes of Transmission (MOT) model was developed to help inform and focus country-specific HIV prevention policies [1, 2]. The MOT – a simple, static, mathematical model – divides the adult population into the following mutually exclusive risk groups: female sex workers (FSW); clients; men who have sex with men (MSM); people who inject drugs (PWID); individuals with casual partners; those at lower risk (i.e. in monogamous partnerships); and the partners of these different risk groups [3]. Parameterization of the model requires data on population sizes, HIV and sexually transmitted infection (STI) prevalence, and sexual behaviour of each risk group to estimate the HIV incidence, and the annual fraction of new HIV infections (FNI) acquired by each risk group. The FNI, which is the main outcome derived with the MOT, is the estimated fraction of all new HIV infections among adults that is acquired by one specific risk group in one year. The MOT results are usually used as part of the wider UNAIDS’ “Know your epidemic, Know your response” synthesis to help allocate HIV prevention resources to the most afflicted risk groups. Before the MOT, the numerical proxy method was often used to help allocate prevention resources. The numerical proxy classifies epidemics as “low-level” or “concentrated” if HIV prevalence remains below 1% in the general population, and remains below or exceeds 5% in a high-risk group, respectively. An epidemic is “generalized” if HIV prevalence among the general population exceeds 1%. With this framework, it is recommended to focus on high-risk groups in concentrated and low-level epidemics, and to target “all segments of society” in generalized epidemics [4, 5].

Although the MOT was designed to improve on the numerical proxy method by quantifying the relative importance of each risk group to the local HIV epidemic [1, 2], concerns have been raised about its utility and its ability to identify the most relevant risk groups for prevention, even in concentrated epidemics [6]. The MOT has been particularly criticized for failing to capture the importance of commercial sex on HIV transmission because of its structural simplicity, variable availability and quality of the data parameterizing the model, and others [7]. Given these concerns, guidelines were published by the HIV Modelling Consortium in 2012 to help improve the use and reporting of the MOT [7].

The objectives of our study are to summarize the MOT syntheses across settings. Our systematic, analytic review adds substantially to the narrative review published by Gouws *et al*. [8] by exploring the sources of variability in MOT results (i.e. by input parameters) across regions and epidemic types, and by evaluating the quality of the MOT studies, as per updated guidelines [7]. In particular, this review assesses the importance of key parameters that reflect behaviour and epidemic setting characteristics on FNI estimates. We also assess the added value of using the largest FNI from the MOT over the numerical proxy method by comparing the potential recommendations that would be or were derived with each method. Our results help determine if the recent guidelines are likely to improve the results of future MOT syntheses, and the utility of the MOT as a tool to inform country-specific HIV intervention programmes.

## Methods

### Search strategy and study selection

We searched the peer-reviewed and grey literature in four stages, conducted according to the criteria of the Preferred Reporting Items for Systematic Reviews and Meta-Analyses group [9]. First, we systematically searched Medline (via PubMed) and EMBASE (via OVID) from 1 January 2003 to 25 September 2012 for published journal articles reporting MOT results using relevant key words (Supplementary file). Titles and abstracts were screened for exclusion followed by full-text review of the remaining studies for inclusion. Second, we compiled a list of UNAIDS countries, and searched websites including UNAIDS, The World Bank, PASCA (Central America HIV/AIDS Prevention Program), and HIV/AIDS Data Hub for Asia and Pacific to identify available UNAIDS MOT reports. For countries without a publically available report, we emailed its UNAIDS country director(s) or the generic UNAIDS “country reporting” team to request the MOT report if one had been conducted. Third, we consulted an “expert panel” (one representative each from The World Bank Global AIDS Program, UNAIDS and Imperial College London, who had been involved in the construction and/or support of the MOT synthesis) to help identify and locate additional and potentially eligible studies. Finally, bibliographies of relevant articles were screened.

### Eligibility criteria

We included MOT studies conducted at the national level. Where multiple MOT syntheses were available for the same country, that is, peer-reviewed and/or UNAIDS reports, we included the most recent one unless two successive MOT analyses were conducted within two years. In these cases, we assessed the quality of both studies but only included the study that provided the most complete quantitative information required for our data analysis.

### Data extraction and analysis

Data was extracted by one investigator (ZS) and verified by another investigator (JV). The quantitative outcomes extracted included the FNI and input parameters on 1) the epidemic setting (HIV prevalence and population size), and 2) behavioural characteristics (annual number of partners, frequency of sex acts and fraction of protected acts), for FSWs, clients, MSM, PWID and the low-risk heterosexual group. We also extracted data on transmission probabilities in the absence of STIs, the prevalence of STI among the aforementioned risk groups and the STI cofactor (the factor by which transmission probabilities are increased in the presence of STI). Where multiple FNI estimates were presented in a study as part of a sensitivity analysis, we extracted the primary estimates and associated input parameters that were reported by the authors of the study (e.g. overall incidence estimates better matching those obtained by a dynamic model). For each risk group, using an approach previously used elsewhere [10, 11], univariate linear regression was used to explore the sources of heterogeneity across FNI estimates due to parameter inputs (as continuous variables) and due to epidemic types – using the reported HIV prevalence among the low-risk group to define a dichotomous, categorical variable (HIV prevalence <1%=low-level and concentrated epidemics, ≥1%=generalized epidemics). For each risk group, we also used multivariate linear regression to assess whether transmission probabilities in the absence of STI, STI prevalence, and STI cofactor could help explain the heterogeneity in FNI between different countries. We present the key associations (i.e. parameters that helped explain the greatest FNI variability) in the main results section and full results in Supplementary file. The analyses were conducted using STATA version 12.0.

MOT study quality was assessed by examining how the MOT was conducted, appraised, and reported, using the eight key recommendations from the 2012 guidelines [7] (Table 1).

**Table 1:** Methods for the assessment of the quality of MOT studies

Recommendations for conducting a high quality MOT	Assessment of quality
Recommendation 1: Synthesize and triangulate available data	We extracted information on the search strategy used by the authors to parameterize the MOT: The authors reported that a systematic review was conducted The search strategy was described in detail and appeared systematic but was not reported as being systematic The authors used multiple sources to locate data to parameterize the MOT The search strategy used was not reported
Recommendation 2: Emphasize the use of the MOT model as a process, that is, where there is insufficient data, use the MOT as a process to help identify gaps in knowledge	We extracted information on: The number of studies that described the MOT exercise as a “process” The key knowledge and data gaps reported by the authors The recommendations made for enhanced surveillance or for further research studies to be conducted in order to address these gaps in knowledge
Recommendation 3: Improve the consideration given to data quality	We extracted information on the main data limitations encountered by the authors.
Recommendation 4: Adopt a bottom-up approach, that is, an approach that ensures that sufficient data is available to parameterize the model before making changes to tailor the MOT to more finely represent the local setting (e.g. by adding additional sub-groups not included in the simple MOT). A “bottom-up” approach involves only tailoring the MOT if there is a need to do so and enough data to parameterize it.	We extracted information on the number of studies that: Amended the MOT by adding sub-groups specific to the local context Used a basic model (did not add sub-groups) Considered tailoring the MOT but judged that it was not possible due to data limitations. Those studies that considered tailoring the model due to a perceived need but instead used a basic model due to data limitations were considered as adopting a “bottom-up approach.”
Recommendation 5: Validate the model results	We extracted information on the number of studies that compared the MOT results with other epidemiological evidence. We included information on what was validated, against what data and the findings.
Recommendation 6: Establish minimum conditions for conducting the MOT analysis	The minimum conditions are not specified in the guidelines. The new EPI-MOT tool [12] has subsequently become available, designed to help countries decide whether the data they have is sufficient to proceed with MOT. We extracted information on whether this tool had been used by countries. Aware that this tool has only recently become available, we retrospectively applied the EPI-MOT to studies, where possible, to find out the number of studies that would have met these “minimum conditions” had the tool been used.
Recommendation 7: Strengthen the uncertainty analysis e.g. extend the uncertainty analysis by allowing for correlated errors, or examining the influence of modelling assumptions on heterogeneities in risk within groups. Present the uncertainty estimates graphically.	We extracted information on: The number of studies that conducted a sensitivity or uncertainty analysis The nature of any sensitivity or uncertainty analyses conducted If the uncertainty was presented graphically
Recommendation 8: Be clear about what the model results mean, that is, the MOT estimates the short-term distribution of infections and does not necessarily reflect the epidemic drivers	We extracted information on whether the authors interpreted the MOT estimated annual fraction of new HIV infections as: The distribution of new infections The source of new infections The driver of the epidemic

We also summarized the key recommendations made by study authors on where to focus HIV prevention resources (high-risk groups, general population or both), and compared this to what would be advocated if using the numerical proxy method or if using the largest FNI.

## Results

### Study inclusions

**Figure 1:**
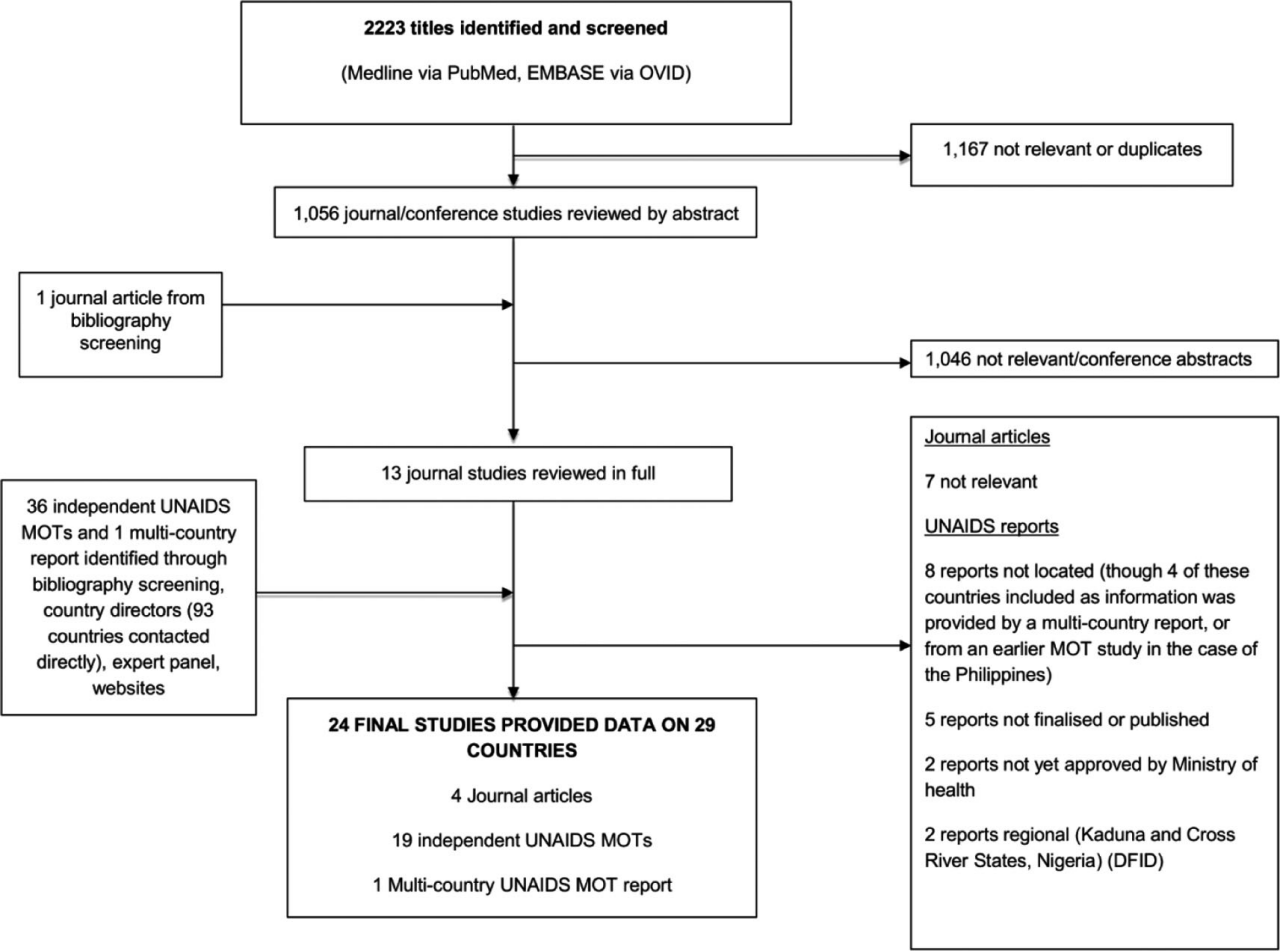
Results of systematic search for eligible studies.

The database search of the published literature yielded 2223 titles, of which two single-country [13, 14] and two multi-country publications [1, 2] met our inclusion criteria and together provided data on five countries with generalized epidemics (Cambodia, Honduras, Kenya, Malawi and Thailand), and three concentrated epidemics (India, Indonesia and Russia) between 2002 and 2010. Pisani’s multi-country MOT (Cambodia, Honduras, Indonesia and Russia) [1], listed above, did not include a low-risk group. Instead, risk groups were restricted to “sex work” (FSW and clients combined), MSM, PWID, those practising casual heterosexual sex, and all the heterosexual partners of high-risk groups combined [1]. We also located 19 UNAIDS MOT studies reporting on a single country from websites and the UNAIDS country teams, covering the Middle East and North Africa (*n=*2) [15, 16], West Africa (*n=*4) [17–20], East and Southern Africa (*n=*6) [21–26], Eastern Europe (*n=*1) [27], Asia (*n=*1) [28], and Latin America and the Caribbean (*n=*5) [29–33]. One West Africa multi-country report [34] provided additional data on Benin, Burkina Faso and Senegal, and complementary information to the UNAIDS MOT studies that reported on Cote D’Ivoire, Ghana and Nigeria [17–19]. At the time of this analysis, Belarus, Guyana, Namibia, Panama and Uzbekistan had postponed their MOT analyses because of insufficient data (personal communication from UNAIDS country teams and [35]). Fourteen MOT analyses were also not included because they had not been completed, published and approved, or the published report was not located despite attempts to contact the respective country team (Supplementary file). All search results and included studies are presented in Figure 1 and Table 2, respectively.

**Table 2:** Summary of included MOT studies

		Assumed HIV prevalence (%)						Risk groups with largest FNI (estimate, minimum-maximum)^c^		Priority group^d^		
				
Country	Year^a^	LRH	FSW	CLIENT	MSM	PWID	Type^b^	1	2	FNI	NP	MOT
Latin America and Caribbean												
Iran [15]	2010	0.04	5	0.5	2.8	15	C	PWID (56, 48–62)%	Part. PWID (12, 10–15)%	HRG	HRG	HRG; GP
Morocco [16]	2010	0.08	2	0.5	2	2	LL	LRH (26, 18–38)%	Client (24, 13–32)%	GP	HRG	HRG
West Africa												
Benin [34]	2009	1	25.5	4.5	10	6	G	LRH (30, 25–35)%	Part. client (15, 13–18)%	GP	HRG; GP	HRG; GP
Burkina Faso [34]	2009	2	21	4	22	6	G	LRH (49, 42–59)%	Part. CHS (11, 8–13)%	GP	HRG; GP	HRG; GP
Cote D’Ivoire [17, 34]	2009	4.5	18.3	13.4	18.5	5.6	G	CHS (32, 25–40)%	LRH (23, 17–29)%	GP	HRG; GP	HRG; GP
Ghana [18, 34]	2008	1.9	37.5	12.3	25.3	5.6	G	LRH (30, 22–36)%	Part. client (22, 19–25)%	GP	HRG; GP	HRG; GP
Nigeria [19, 34]	2009	3.6	34	10.8	13.5	5.6	G	LRH (42, 30–45)%	Part. CHS (15, 12–18)%	GP	HRG; GP	HRG; GP
Senegal [34]	2009	0.5	19.5	2	22	2	C	Part. CHS (35, 25–45)%	CHS (22, 10–32)%	GP	HRG	HRG; GP
Sierra Leone [20]	2010	1.2	8.5	1.5	7.5	4	G	Clients (26%, nc)	LRH (16%, nc)	HRG	HRG; GP	HRG; GP
East and Southern Africa												
Kenya [2]	2005	7.5	40	8.1	20	20	G	LRH (30%, nc)	Part. CHS (28%, nc)	GP	HRG; GP	HRG; GP
Kenya [21]	2006	7.4	NS	NS	NS	NS	G	Part. CHS (28%, nc)	CHS (20%, nc)	GP	HRG; GP	HRG; GP
Lesotho [22]	2008	23.2	NS	NS	NS	NA	G	LRH (35, 35–62)%	CHS (31, 16–31)%	GP	HRG; GP	GP
Malawi [14]	2008	13	70.7	17	20	NA	G	LRH (37%, nc)	Part. CHS (27%, nc)	GP	HRG; GP	GP
Swaziland [23]	2008	33	60	45	40	25.9	G	LRH (50, 48–65)%	Part. CHS (21, 12–21)%	GP	HRG; GP	GP
Uganda [24]	2008	5	47.2	8.5	43	30	G	LRH (43, 41–46)%	CHS (24, 21–27)%	GP	HRG; GP	HRG; GP
Zambia [25]	2008	14.3	68.7	39	33	NA	G	Part. CHS (37%, nc)	CHS (34%, nc)	GP	HRG; GP	HRG; GP
Zimbabwe [26]	2010	14.3	54.3	19.3	16.8	12.4	G	LRH (55, 50–68)%	Part. CHS (14%, nc)	GP	HRG; GP	HRG; GP
Eastern Europe and Russia												
Moldova [27]	2010	0.1	6.8	1.3	0.7	17.8	C	Part. PWID (31, 18–51)%	Part. CHS (16, 5–28)%	GP	HRG	HRG; GP
Russia [1]	2002	NS	NS	NS	NS	NS	C	PWID (61%, nc)	Part. HRG (25%, nc)	HRG	HRG	HRG^e^
ASIA												
Cambodia [1]	2002	NS	NS	NS	NS	NS	G	Part. HRG (56%, nc)	FSW & clients (24%, nc)	GP	HRG; GP	HRG; GP
India [13]	2010	0.3	4.9	1	7.3	9.2	C	LRH (63%, nc)	PWID (14%, nc)	GP	HRG	NA
Indonesia [1]	2002	NS	NS	NS	NS	NS	C	PWID (82%, nc)	FSW & clients (9.5%, nc)	HRG	HRG	HRG^e^
Philippines [28]	2010	0	0.2	0.01	1	0.06	LL	MSM (89%, nc)	OPW (7%, nc)	HRG	HRG	HRG^e^
Thailand [2]	2005	0.6	5	5	7	45	C	LRH (43%, nc)	MSM (21%, nc)	GP	HRG	HRG; GP
Latin America and Caribbean												
Dominican Republic [29]	2010	0.8	4.8	2.2	6.1	12.8	C	MSM (33, 23–45)%	LRH (32, 23–38)%	HRG	HRG	HRG^e^; GP^e^
El Salvador [30]	2011	0.3	4.1	2	9.8	3	C	MSM (36, 27–44)%	Client (15, 8–30)%	HRG	HRG	HRG^e^
Honduras [1]	2002	NS	NS	NS	NS	NS	G	MSM (40%, nc)	Part. HRG (36%, nc)	HRG	HRG; GP	HRG^e^; GP^e^
Jamaica [31]	2012	1.1	4.4	3.5	15	NA	G	MSM (32, 25–40)%	CHS (22, 15–30)%	HRG	HRG; GP	HRG; GP
Nicaragua [32]	2011	0.6	1.9	1	7.5	1.9	C	MSM (44, 25–52)%	CHS (21, 16–38)%	HRG	HRG	HRG^e^; GP^e^
Peru [33]	2010	0.3	0.9	0.8	5.2	13	C	MSM (55, 35–66)%	LRH (16, 5–22)%	HRG	HRG	HRG^e^

a Year of MOT estimate;

b Epidemic type (LL=low-level, C=concentrated, G=generalized);

c Where sensitivity or uncertainty analysis is conducted, the minimum and maximum estimates are provided. Where such analyses were not conducted only the estimate is provided (nc=not conducted);

d Priority risk groups for prevention resources (FNI=group based on largest fraction of new HIV infection, NP=group based on numerical proxy method, MOT=actual recommendations made by each MOT study author);

e No group specifically specified by the country MOT authors but authors recommended that resources should be based on MOT outputs – here assumed based on the top group±second group if estimated FNI is within 10% of the top risk group and/or overlapping confidence intervals. For example, if the authors did not explicitly say that MSM should be prioritized but instead said that resources should be aligned with the MOT results and MSM acquired the largest FNI, MSM here are considered to have been prioritized; CHS=casual heterosexual sex; FSW=female-sex worker; HRG=high-risk groups; GP=general population (includes bridging populations); LRH=low-risk heterosexual adult; MSM=men who have sex with other men; NA=not applicable (e.g. no specific groups prioritized, group excluded or risk groups not disaggregated); NS=not specified; OPW=overseas Philippino worker; Part=partner; PWID=people who inject drugs; SA=sensitivity analysis.

### Variability in the FNI among high-risk groups across regions and epidemic types

**Figure 2:**
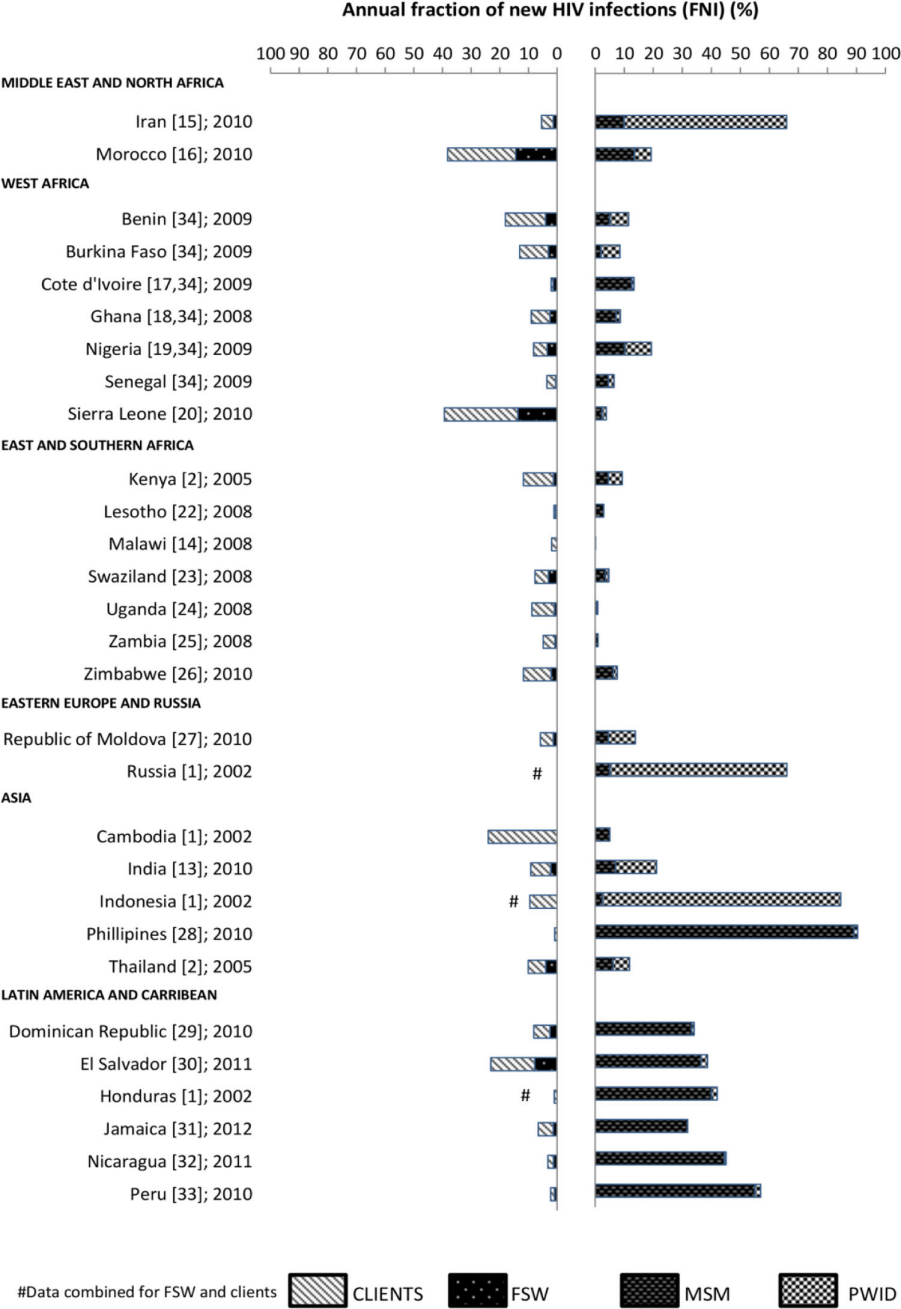
The FNI among high-risk groups by region.

The FNI for high-risk groups by region are shown in Figure 2. The FNI among FSWs was highest in Morocco (FNI=14.4%), Sierra Leone (13.7%) and El Salvador (7.8%) compared to 4% or less (median 1.3%) in the remaining 23 countries, including India (2.2%) and Thailand (4.0%), where FSWs play an important role in HIV transmission [36, 37]. Similarly, the FNI among clients was highest in Sierra Leone, Morocco, El Salvador and Benin with 25.6, 23.8, 15.3, and 14.0%, respectively, and varied between 0 and 10.5% (median 4.6%) across the remaining 22 countries. Overall, the FNI was generally higher among MSM (median 7%) than FSWs (median 1.3%) and clients (median 5%), especially in Latin America and the Caribbean (median MSM 38.2%), and the Philippines (MSM 89.2%). The FNI among PWID was relatively low and homogenous across countries (median 1.5%, range 0 to 14.1%), except Indonesia, Iran and Russia, where it was very high (82.0, 61.0, and 56.0%, respectively). However, Indonesia’s and Russia’s MOT (part of Pisani’s study [1]) did not include infections from the low-risk group in their denominator of the total number of HIV infections meaning that the estimated contribution of PWID will be larger than other countries. These estimates, therefore, may not be directly comparable with countries that did include the low-risk group.

**Figure 3:**
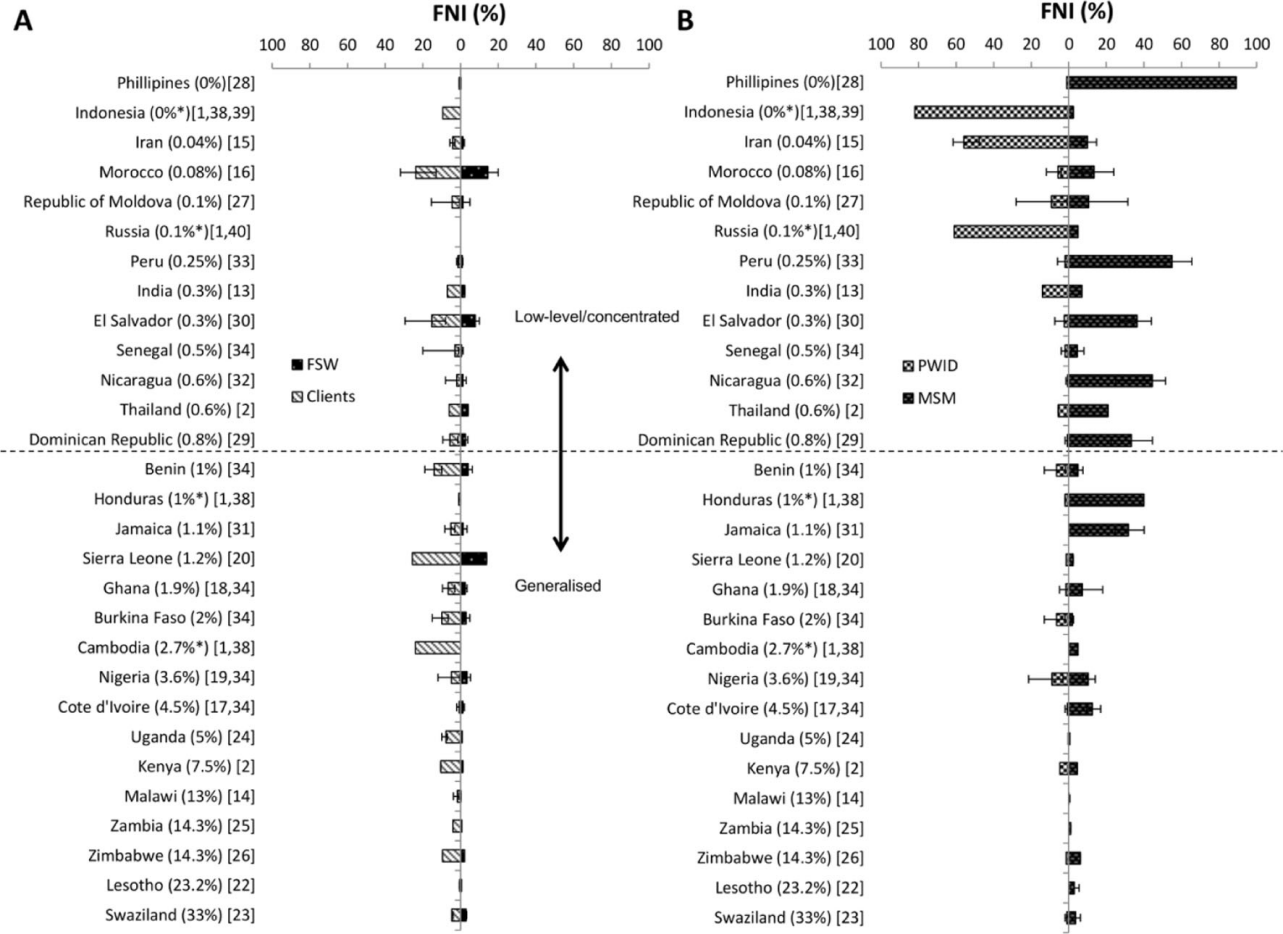
The FNI among high-risk groups by the assumed HIV prevalence in the low-risk group.

Figure 3a and 3b shows the FNI among FSWs and clients, and among MSM and PWID, ranked by the reported HIV prevalence in the low-risk group, respectively.

We have used the assumed HIV prevalence among the low-risk group to categorize studies (<1%=low-level and concentrated epidemics, ≥1%=generalized epidemics). Although Pisani’s 2002 MOT study (Indonesia, Russia, Honduras and Cambodia) did not include a low-risk group, we used the HIV prevalence among pregnant women or the low range adult HIV prevalence as a “proxy” for epidemic type for illustrative purposes only; these values are not used for the regression analyses. *denotes that FSW and client FNI are combined. The error bounds relate to the sensitivity or uncertainty analysis carried out in each MOT study, where applicable, and denotes the minimum and maximum values.

The FNI among FSWs and clients was similar between low-level or concentrated epidemics (FSW=1.3%, clients=4.3%, median) and generalized epidemics (FSW=1.7%, clients=5.9%, median) (FSW *R*^2^=0.00, *p=*0.83; Client *R*^2^=0.01, *p=*0.59). In contrast, the FNI among MSM was almost three times larger in low-level and concentrated epidemics (median 13.5%) than generalized epidemics (median 4.7%) (*R*^2^=0.18, *p=*0.02). Similarly, the median FNI among PWID was higher in low-level and concentrated (median 5.7%) than generalized epidemics (median 1.3%) (*R*^2^=0.16, *p=*0.03).

### Sources of heterogeneity in the FNI among high-risk groups

Excluding Sierra Leone, Morocco and El Salvador, the FNI among FSWs was uniformly low (≤4% in 23 countries) across settings and epidemic types despite variability in model parameters: FSW population size (range, 0.3 to 3.2% of adult females); HIV prevalence among FSW (0.2 to 70.7%) and clients (0.01 to 45%); STI prevalence among clients (0.05 to 27.7%); yearly FSW client volume (42 to 843); annual number of sexual acts per client (1 to 9). However, condom use among FSWs in these 23 countries was consistently high (median 73%, range 50 to 92%) compared to the three countries with the largest FNI among FSWs [Sierra Leone (17%), Morocco (25%), El Salvador (52%)], and similarly for clients (data not shown). Condom use was strongly and negatively associated with the FNI among FSWs (*R* ^2^=0.55, *p*≤0.001) and clients (*R* ^2^=0.46, *p*≤0.001).

**Figure 4:**
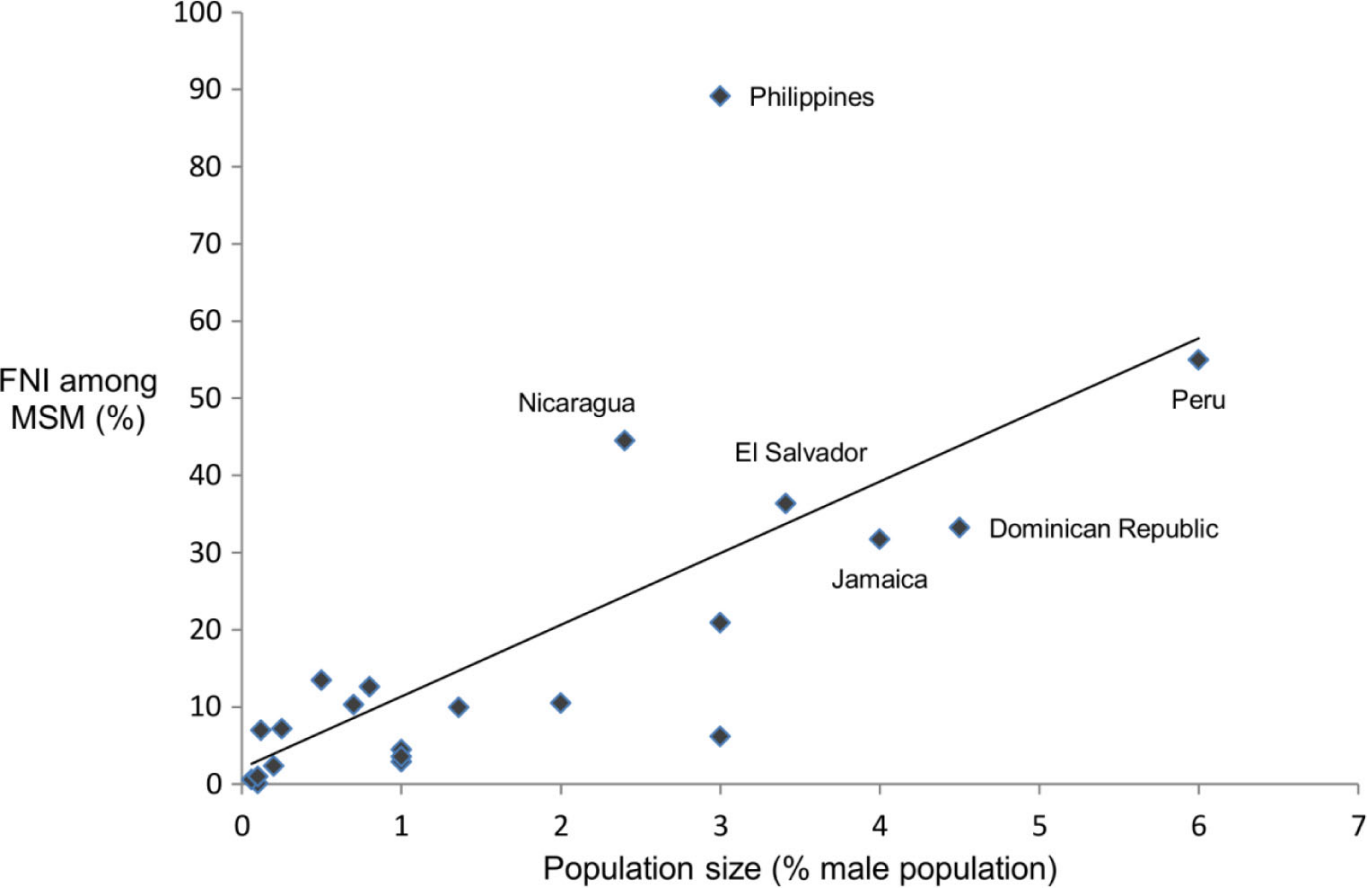
The FNI among MSM versus their population size.

Among MSM, the relative size of the MSM population was positively associated with the FNI (*R*^2^=0.50, *p*≤0.001) (Figure 4).

In all studies (excluding two studies not reporting this information [1, 34]), the population size of PWID was universally small (median 0.05%, range 0.0 to 1.3% of the total population), and rather homogeneous between concentrated (median 0.06%) and generalized (median 0.05%) epidemics. Despite the small population size of PWID, however, we observed a relatively large FNI among PWID in some concentrated epidemic settings. The FNI among PWID was positively correlated with the HIV prevalence ratio of PWID to the low-risk group (*R* ^2^=0.81, *p*≤0.001), which explained much of the variability across epidemic types. For example, in Iran and India, with the third and fourth largest FNI among PWID (56 and 14%), the assumed HIV prevalence among PWID (15.0 and 9.2%) was nearly 400 and 30 times that of the low-risk population (0.04 and 0.3%), respectively. In contrast, the smallest FNI among PWID were observed in countries such as Swaziland and Zimbabwe, where the HIV prevalence in PWID was assumed to be lower than in the low-risk group (HIV prevalence ratios of 0.8, 0.9, respectively). Indonesia and Russia had the two largest FNI among PWID because the low-risk population was excluded from the model, which is the equivalent of setting the HIV prevalence in the low-risk group to zero.

There was little variability between MOT studies in terms of the transmission probabilities in the absence of STI and the STI cofactor (ranges provided in Supplementary file), with most countries using the built-in MOT default values. These parameters as well as STI prevalence, the number of partners per year, and the number of acts per partner per year did not explain the variability between country MOT outputs (Supplementary file).

### Sources of heterogeneity in the FNI among the low-risk group

**Figure 5:**
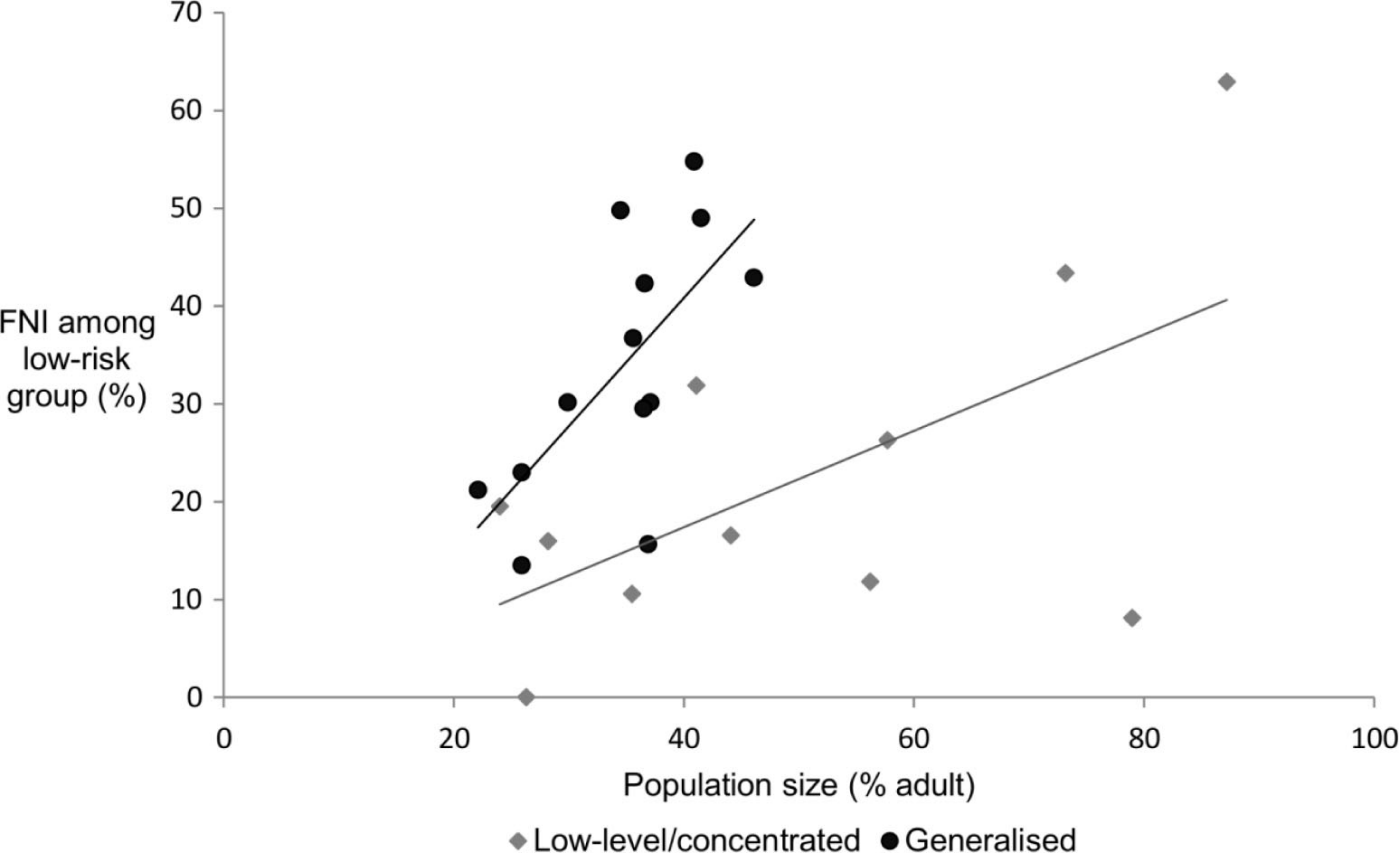
The FNI among the low-risk group versus their population size.

Twenty-two out of twenty-five MOT studies that included a low-risk group in their MOT model estimated that the low-risk group was one of the three risk groups with the largest FNI (low-risk group median FNI 30.2%, range 11.8 to 62.9%) (data not shown). In 13 countries, the largest FNI was acquired by the low-risk group (Table 2), including countries with low-level or concentrated epidemics such as Morocco, India and Thailand, where 26.3, 62.9 and 43.4% of new infections were acquired by the low-risk group, respectively. The FNI among the low-risk group, who are generally the largest risk group with a median population of 36.8% of the adult population, was positively correlated with their population size, particularly in generalized epidemics (*R* ^2^=0.47) (Figure 5).

### Qualitative assessment

The results of the MOT quality assessments (as per recommendations described in Table 1) are summarized in Table 3.

**Table 3:** Quality of the conducted MOT studies

Country	Search; R1	Process; R2	Reported data gaps^a^; R2/3	Recommend research based on data gaps; R2/3	Other groups considered; R4	Model; R4	Validation; R5	EPI-MOT; R6	UA or SA^b^; R7	Interpretation^c^; R8
Middle East and North Africa										
Iran [15]	SR	No	Yes	Yes	Yes	BM	Yes	No^d^	Yes	Distribution
Morocco [16]	SR	Yes	Yes	Yes	Yes	BM	Yes	No^d^	Yes	Distribution, driver^e^
West Africa										
Benin [34]	MS	No	Yes	Yes	No	BM	Yes	No	Yes	Distribution
Burkina Faso [34]	MS	No	Yes	Yes	No	BM	Yes	No	Yes	Distribution
Cote D’Ivoire [17, 34]	MS	No	Yes	Yes	No	BM	Yes	No^d^	Yes^f^	Distribution
Ghana [18, 34]	MS	No	Yes	Yes	No	BM	Yes	No^d^	Yes^f^	Distribution
Nigeria [19, 34]	MS	Yes	Yes	Yes	Yes	BM	Yes	No^d^	Yes^f^	Distribution
Senegal [34]	MS	No	Yes	Yes	No	BM	Yes	No	Yes	Distribution
Sierra Leone [20]	MS	Yes	Yes	Yes	Yes	CM (AG)	Yes	No^d^	No	Distribution, driver^e^
East and Southern Africa										
Kenya [2]	NS	No	No	Yes	No	BM	Yes	No	No	Distribution
Kenya [21]	MS	No	Yes	Yes	Yes	CM (AG)	No	No	No	Distribution
Lesotho [22]	MS	Yes	Yes	Yes	Yes	BM (EG)	Yes	No	Yes	Distribution, driver^e^
Malawi [14]	NS	No	No	No	Yes	BM (EG)	Yes	No^d^	Yes	Distribution
Swaziland [23]	MS	Yes	Yes	Yes	Yes	BM	Yes	No^d^	Yes	Distribution, driver^e^
Uganda [24]	MS	Yes	Yes	Yes	Yes	BM	Yes	No	Yes	Distribution
Zambia [25]	MS	Yes	Yes	No	Yes	BM (EG)	Yes	No^d^	No	Distribution
Zimbabwe [26]	MS	Yes	Yes	No	Yes	BM	Unclear	No	Yes	Distribution, driver^e^
Eastern Europe and Russia										
Moldova [27]	SR	No	Yes	Yes	Yes	BM	Yes	No^d^	Yes	Distribution
Russia [1]	NS	No	No	No	No	BM	No	No	No	Distribution
Asia										
Cambodia [1]	NS	No	No	No	No	BM	No	No	No	Distribution
India [13]	MS	No	No	No	No	BM	Yes	No^d^	Yes	Distribution
Indonesia [1]	NS	No	No	No	No	BM	No	No	No	Distribution
Philippines [28]	MS	No	Yes	Yes	Yes	CM (AG, EG)	Yes	No^d^	No	Distribution
Thailand [2]	NS	No	No	Yes	No	BM	Yes	No	No	Distribution
Latin America and Caribbean										
Dominican Republic [29]	MS	Yes	Yes	Yes	Yes	CM (AG)	No	No^d^	Yes	Distribution
El Salvador [30]	MS	Yes	Yes	Yes	Yes	BM	No	No^d^	Yes	Distribution
Honduras [1]	NS	No	No	No	No	BM	No	No	No	Distribution
Jamaica [31]	MS	Yes	Yes	Yes	Yes	BM (EG)	Yes	No^d^#	Yes	Distribution, driver^e^
Nicaragua [32]	MS	Yes	Yes	Yes	Yes	CM (AG)	No	Yes	Yes	Distribution
Peru [33]	MS	Yes	Yes	Yes	No	BM	No	No^d^	Yes	Distribution

a Further details on the data limitations encountered by studies are provided in Supplementary file;

b Further details on the methods used for these analyses and their results can be found in Supplementary file;

c Interpretations of MOT annual fraction of new HIV infections (FNI) (Distribution=the annual fraction of new infections, Source=the source of new infections, Driver=the drivers of the epidemic);

d passed Part1 EPI-MOT [12] when tool retrospectively applied;

e Lesotho, Sierra Leone, Swaziland, Uganda and Zimbabwe included any contextual, social or structural factors that increase the risk of HIV transmission into their definition of “driver,” Jamaica defined “driver” using a “R0 analysis” and Morocco defined high-risk groups as the “drivers” and not the low-risk population who had the largest FNI;

f Uncertainty analyses conducted later as part of the West Africa multi-country report; AG=additional risk groups added; BM=basic model; CM=customized model; EG=excluded risk groups that were deemed not important in the local context. This was not defined as a customisation but rather the application of the basic model with the population size of the relevant group set to zero; MS=multiple sources; R1–R8=recommendations 1 to 8 based on Case *et al*. guidelines [7]; SA=sensitivity analysis; SR=systematic review; UA=uncertainty analysis.

All UNAIDS country MOT reports but only one (1/4) MOT journal article [13] synthesized and triangulated multiple data sources (Recommendation 1). Three country studies (3/30) performed a systematic review to inform MOT parameters [15, 16, 27], and six studies (6/30) detailed their comprehensive search strategy [20, 22–26].

Most UNAIDS reports adequately described the MOT exercise as a “process” [16, 19, 20, 22–26, 29–33] (13/22) (Recommendation 2), acknowledging data limitations, particularly for high-risk groups (Supplementary file), and recommending enhanced surveillance and additional epidemiological research to address the identified key data gaps (Tables 2 and 3, and Supplementary file).

Although 16 UNAIDS MOT reports considered other sub-groups as potentially important to their local epidemics, most (11/16) did not alter the model because of data limitations, and, thus, adopted the recommended “bottom-up” approach (Recommendation 4). Five countries (5/30) customized the MOT model to local settings [20, 21, 28, 29, 32] by adding additional or disaggregating groups. For example, the Philippines disaggregated FSWs into registered and freelance sex workers [28]. Uganda undertook an additional sub-analysis examining age and sex distributions of infections among individuals practising casual and monogamous sex [24].

Thirteen MOT studies [2, 16–20, 22–25, 27, 31] (13/30) attempted to validate their results (Recommendation 5) by comparing the MOT’s total annual number of new infections to estimates from a dynamic model such as Spectrum [42] or the Asian Epidemic Model [43]; the MOT estimates tended to be lower than Spectrum’s (Supplementary file). Other studies validated the MOT results by comparing either the MOT’s estimates of overall prevalence in the adult population to those of Spectrum’s [15] or the MOT’s estimates of overall incidence in the adult population to those obtained from the Demographic Health Survey [14] or national surveillance data and the HIV Registry [28]. The West Africa multi-country report [34] used national country-specific data that provided a “plausibility range” of the total annual number of new infections among adults, and allowed only those parameter combinations that resulted in a number falling within this range. Although the MOT framework includes built-in “checks” that ensure that inputted parameters balance (e.g. the total number of commercial acts carried out by all FSWs must match the total number of commercial acts carried out by all male clients), this does not guarantee that these input parameters are plausible. In Sierra Leone’s MOT study [20], for example, each client was assumed to perform almost three commercial acts with a FSW per day in order to match-up FSW and client data. A similarly high number of visits to FSWs was assumed in El Salvador’s [30] analysis, which was one commercial act with a FSW every three days.

Despite mentioning the need to establish the “minimum conditions” before conducting the MOT, these conditions are not specified in the guidelines [7] (Recommendation 6). However, UNAIDS introduced the EPI-MOT [12] tool in 2012 to help countries decide if sufficient data is available to conduct a MOT synthesis. The first stage of the EPI-MOT assesses data availability for all parameters for high-risk groups, those practising casual sex and those in monogamous partnerships. A country “passes” stage 1 if there is enough data to inform 50% of MOT parameters, with greater importance given to population size and HIV prevalence (these parameters are given double the weight compared to other parameters). Stage 2 assesses the quality of the available data. Recent, regional and representative data of the population modelled, for example, are considered of good quality. To date, only one country has used and successfully passed the EPI-MOT [32]. Thus, we retrospectively applied stage 1 to all 17 country reports that reported whether data was available for the required MOT parameters [13–20, 23, 25, 27–33] , and found that all studies would have “passed” this first stage. Due to inconsistent reporting of the data sources and the use of data from unpublished or grey literature we were unable to retrospectively apply stage 2 of the EPI-MOT. However, many studies reported the use of data of sub-optimal quality (e.g. outdated non-regional estimates or based on expert opinion only), particularly for high-risk groups [15, 16, 18–20, 22–31, 33, 34] (Recommendation 3). In order to better appreciate the utility of the EPI-MOT, we performed a variety of EPI-MOT “mock” exercises and found that countries could meet the EPI-MOT’s “minimum conditions” even if no empirical data on the population size and HIV prevalence of all risk groups was available, relying solely on assumptions instead. This is despite these parameters having twice the weight of other parameters because a country only needs to gain 50% of the overall marks to pass Part 1 of the EPI-MOT (which is possible without information on population size and HIV prevalence). Although some countries have postponed the MOT because of insufficient data (Belarus, Namibia and Uzbekistan), it is unclear if this was based on EPI-MOT results. Guyana’s and Panama’s MOT country teams did, however, utilize the EPI-MOT and decided to postpone their studies because of data limitations (personal communication, J. Vesga, and [35]).

Twenty country studies performed a sensitivity or uncertainty analysis to parameter assumptions (20/30), and 17 reported this uncertainty in the presentation of results (Table 3 and Supplementary file). None “strengthened” their uncertainty analysis by examining the influence of potential correlations between parameters or of structural assumption (Recommendation 7).

All MOT studies correctly interpreted the MOT results as the one-year percentage distribution or FNI rather than as the source of these new HIV infections [44] (i.e. drivers of transmission) (Recommendation 8). Eight country studies (8/30) discussed epidemic “drivers” in their MOT reports but not exclusively related to the MOT FNI [16, 20, 22–24, 26, 27, 31], with Lesotho, Sierra Leone, Swaziland, Uganda and Zimbabwe, including contextual, social or structural factors that increase the risk of HIV transmission into their discussion of epidemic “drivers” [20, 22–24, 26]. Jamaica calculated the number of secondary HIV cases from all infected individuals within each risk group to identify the epidemic “drivers” of transmission [31]. The Republic of Moldova identified their PWID population as being the key “driver” of the HIV epidemic not based on the FNI but because PWID had the highest HIV prevalence [27]. Morocco identified high-risk groups as the epidemic “drivers” despite the low-risk group acquiring the largest FNI [16].

### Reports’ recommendations on resource allocation following the MOT synthesis

Twenty-one MOT syntheses (21/30) made specific recommendations on the type of interventions to implement and where to focus efforts based on their epidemiological review and the FNI. Eight countries did not specify which specific risk-group should be prioritized but suggested that prevention resources should be aligned with the FNI (Table 1). In most countries (*n=*20), recommendations by the MOT authors on which risk groups (high-risk, general population, or both) should be prioritized for prevention were the same as those that would have been reached using the simpler numerical proxy method. For example, in Morocco, a low-level epidemic setting, the low-risk group had the largest FNI. However, the authors recommended focusing prevention on all high-risk groups, in line with recommendations advocated if using the numerical proxy method. In Sierra Leone’s generalized epidemic, the authors made key recommendations on nearly 20 groups including those not evaluated in the MOT, which is also in line with recommendations that would be derived using the numerical proxy method, that is, focus on “all segments of society.”

## Discussion

The MOT was designed to help focus country-specific HIV prevention policies [2]. We conducted a systematic and analytic review of national MOT studies to assess the utility of the MOT.

We found that the FNI among MSM and PWID varied between regions or epidemic types. However, the FNI among FSWs and clients was homogeneously low across most regions and epidemic types, and among the low-risk population was large in most countries. Most MOTs are being conducted and reported as per guidelines but data quality remains an issue. Our results also suggest that the MOT is not necessarily more informative than the numerical proxy method. Our findings raise some concerns about the utility of the FNI for allocating HIV prevention resources.

The universally low FNI among FSWs was partly explained by assumptions of high condom use across studies, likely because of existing prevention efforts among FSWs and their clients [34]. A large FNI among PWID was restricted to concentrated epidemics, where HIV prevalence among PWID was much larger than in the low-risk group. In many countries, including those with low-level or concentrated epidemics, the largest FNI was among the low-risk population. If the FNI is used to guide the allocation of prevention resources, there is a risk that resources could be re-allocated to the large low-risk group and away from high-risk groups who are more likely “driving” the epidemic. This is less cost-effective and could potentially reverse the positive impact of existing interventions.

Using the FNI in combination with the HIV incidence rate estimates among risk groups may reduce potential misinterpretation about the practical implications of large FNI estimates among the low-risk population, particularly in low-level and concentrated epidemics. Refining the model structure may also help reduce concerns about the underestimation of the contribution of high-risk groups to the local HIV epidemic. Indeed, a recent study by Prudden *et al*. [45], suggested that the FNI acquired by high-risk groups was more than double of that estimated by the original MOT when the low-risk population are disaggregated into monogamous sero-discordant and sero-concordant couples, and the latter re-categorized as “very low-risk” (i.e. not contributing any new infections). This essentially amounted to reducing the size of the low-risk population and the denominator of total infections, similar to Pisani’s MOT study [1].

The correlation between the FNI acquired by MSM and the low-risk population and their respective population size highlights the sensitivity of the FNI to population size. The population size of the low-risk group in the MOT is derived by subtracting the sum of the population sizes of all other risk groups from 100%, underscoring the need for reliable population size data for all risk groups. Importantly, this suggests that high-risk populations such as MSM, who are often hidden and their size underestimated, may be underrepresented by the MOT.

The majority of MOT studies acknowledged data limitations, particularly for high-risk groups. The EPI-MOT tool [12] may help countries decide if the available data are sufficient to conduct an MOT, and to identify data gaps. We found that all MOT studies on which we retrospectively applied Part 1 of the EPI-MOT “passed” this first part. Nevertheless, the EPI-MOT criteria were found to be insufficient to “fail” a country at stage 1 even without data on the population sizes and HIV prevalence of all risk groups. It is advised to postpone the MOT if a country fails the EPI-MOT. Yet if a country passes the EPI-MOT it does not necessarily mean that their data is adequate as shown in the Sierra Leone MOT [20] which assumed implausible number of FSW visits by clients in order to equalize the number of client acts to FSW acts, due to the assumed small client population. Prudden *et al*. noted that Nigeria’s Cross River State regional MOT study had not equalized the sex acts between one population sub-group and their partners [45]. We recommend that the EPI-MOT minimum conditions be revised and validated to establish sufficiently sensitive and specific criteria that ultimately improve the use and interpretation of MOT results.

Although many MOT studies tried to validate their results, this only involved validating total population HIV incidence or prevalence. To improve FNI estimates, efforts should be made to also validate HIV incidence estimates by risk group, and to include a mechanism for assessing the plausibility of key parameter values. The MOT should, as far as possible, be calibrated and the data triangulated and contextualized. However, with a model like the MOT, there is a limit to the amount of validation that can be done and what can be fitted, unlike transmission dynamic models that can make use of more time series data, for example.

MOT analyses reporting varied between studies. Some did not provide a complete list of input parameters thus precluding them from our quantitative analyses. Future MOT studies should report all parameters, their sources and their justifications, as per good HIV epidemiology modelling practice [46]. All UNAIDS country reports triangulated data while only journal article (1/4) did so.

Another “weakness” of the MOTs to date is the limits of the uncertainty analyses. The built-in sensitivity analysis tool does not easily allow the user to take into account potential correlations between parameters or to assess the sensitivity of the results to structural assumptions, particularly for those less experienced in modelling. Although we advocate the use of uncertainty estimates when reporting MOT results, when uncertainty estimate ranges are relatively large, as in the example of Moldova’s MOT study, the results will be imprecise and thus potentially uninformative. This large uncertainty in MOT results will be largely due to poor quality data used to parameterize the model. In such cases, it may be wise to postpone the MOT until better quality data is available rather than attempt to interpret the results for the allocation of HIV prevention interventions.

We found that many countries highlighted multiple or all risk groups to be the focus of HIV prevention resources. Although this may be a necessary political strategy, advocating the focus of resources on “all segments of society” does not necessarily coincide with the original objectives of the MOT, that is, to help guide country-specific and focused allocation of HIV prevention resources.

We found that in most countries, recommendations by the authors on which risk groups should be prioritized for prevention were often similar to those that would have been made using the simpler and highly criticized numerical proxy method. Nevertheless, we are not advocating returning to the numerical proxy method. Instead we recommend that the modelling be improved, producing valid recommendations. An improved model would address some of the limitations identified with the MOT model [7, 13, 44, 45, 47]. The FNI provides short-term estimates on who is acquiring infection rather than long-term estimates on who is contributing the most to transmission. Thus, the FNI should not be interpreted as the “source” of HIV infections [44]. Furthermore, the short-term static nature of the model does not allow the tracking of infections. For example, those that are categorized as low-risk may have acquired their infection a few years previously when they were sex workers. Indeed, both the numerical proxy method and the MOT have been shown to underestimate the contribution of the epidemic drivers [13, 47]. Having different models for different epidemic types may be a potential option; though this would not be entirely satisfactory as it would require to first determine the true epidemic type in order to determine which model would be appropriate to use for subsequent analysis [47]. This is slightly paradoxical because determining the epidemic type requires that the epidemic drivers are known; this is the information that we want to derive from our modelling tool. Ideally, a carefully calibrated dynamic model should be used because it has the ability to produce estimates of the FNI and to define the drivers of the epidemic and the epidemic type, taking into account the long-term contribution of transmission.

## Conclusions

Although countries are generally performing the MOT as per recent guidelines, results showed little variation in MOT results (except MSM and PWID) by regions and epidemic types. Homogeneity in MOT outputs for FSWs, clients and the low-risk population may limit the utility of MOT for guiding country-specific interventions in heterosexual HIV epidemics. Although the new EPI-MOT tool may be a useful tool to improve data quality, it is recommended that its minimum conditions for proceeding with a MOT exercise be revised.

## References

[1] Pisani E, Garnett GP, Brown T, Stover J, Grassly NG, Hankins C (2003). Back to basics in HIV prevention: focus on exposure. BMJ.

[2] Gouws E, White PJ, Stover J, Brown T (2006). Short term estimates of adult HIV incidence by mode of transmission: Kenya and Thailand as examples. Sex Transm Infect.

[3] Joint United Nations Programme on HIV/AIDS (UNAIDS) (2007). Modelling the expected short-term distribution of incidence of HIV infections by exposure group. http://www.unaids.org/en/dataanalysis/tools/incidencebymodesoftransmission.

[4] Joint United Nations Programme on HIV/AIDS (UNAIDS) (2005). Intensifying HIV prevention: UNAIDS policy position paper. http://data.unaids.org/publications/irc-pub06/jc1165-intensif_hiv-newstyle_en.pdf.

[5] Joint United Nations Programme on HIV/AIDS (UNAIDS), World Health Organization (WHO) (2000). Second generation surveillance for HIV: the next decade. http://whqlibdoc.who.int/hq/2000/WHO_CDS_CSR_EDC_2000.5.pdf.

[6] The HIV Modelling Consortium. Sources of infections and national intervention impact projections (2011). http://www.hivmodelling.org/sites/default/files/uploads/documents/meeting-reports/Meeting%20Report%201%20-%20Montreux.pdf.

[7] Case KK, Ghys PD, Gouws E, Eaton JW, Borquez A, Stover J (2012). Understanding the modes of transmission model of new HIV infection and its use in prevention planning. Bull World Health Organ.

[8] Gouws E, Cuchi P, International Collaboration on Estimating HIV Incidence by Modes of Transmission (2012). Focusing the HIV response through estimating the major modes of HIV transmission: a multi-country analysis. Sex Transm Infect.

[9] Moher D, Liberati A, Tetzlaff J, Altman DG, PRISMA Group (2009). Preferred reporting items for systematic reviews and meta-analyses: the PRISMA statement. PLoS Med.

[10] Mishra S, Steen R, Gerbase A, Lo YR, Boily MC (2012). Impact of high-risk sex and focused interventions in heterosexual HIV epidemics: a systematic review of mathematical models. PLoS One.

[11] Pickles M, Boily MC, Vickerman P, Lowndes CM, Moses S, Blanchard JF (2013). Assessment of the population-level effectiveness of the Avahan HIV-prevention programme in South India: a preplanned, causal-pathway-based modelling analysis. Lancet Glob Health.

[12] Joint United Nations Programme on HIV/AIDS (UNAIDS) (2012). Epidemiological review related to the Modes of Transmission analysis (Epi–MoT). http://www.unaids.org/en/media/unaids/contentassets/documents/document/2012/guidelines/JC2428_Epireview_ModesofTransmissionAnalysis_en.pdf.

[13] Mishra S, Sgaier SK, Thompson LH, Moses S, Ramesh BM, Alary M (2012). HIV epidemic appraisals for assisting in the design of effective prevention programmes: shifting the paradigm back to basics. PLoS One.

[14] Maleta K, Bowie C (2010). Selecting HIV infection prevention interventions in the mature HIV epidemic in Malawi using the mode of transmission model. BMC Health Serv Res.

[15] Joint United Nations Programme on HIV/AIDS (UNAIDS), Kerman University of Medical Sciences (Knowledge Hub for HIV/AIDS surveillance) (2011). Modeling of new HIV infections based on exposure groups in Iran.

[16] Joint United Nations Programme on HIV/AIDS (UNAIDS) (2010). Ministère de la santé, DELM/PNLS, Morocco, Infectious Disease Epidemiology Group, Weill Cornell Medical College, Qatar. HIV modes of transmission analysis in Morocco.

[17] Joint United Nations Programme on HIV/AIDS (UNAIDS) (2009). Modes de transmission du VIH en Côte d’Ivoire: Analyse de la distribution des nouvelles infections par le VIH en Côte d’Ivoire et la recommandations pour la prevention.

[18] Joint United Nations Programme on HIV/AIDS (UNAIDS) Ghana AIDS Commission (2009). Modes of HIV transmission in West Africa: analysis of the distribution of new HIV infections in Ghana and recommendations for prevention.

[19] Joint United Nations Programme on HIV/AIDS (UNAIDS), The World Bank (2009). Modes of HIV transmission in Nigeria: analysis of the distribution of new HIV infections in Nigeria and recommendations for prevention.

[20] Joint United Nations Programme on HIV/AIDS (UNAIDS) (2010). UNICEF, National AIDS Secretariat, Sierra Leone. Sierra Leone HIV Modes of Transmission Study: know your epidemic, know your response.

[21] Joint United Nations Programme on HIV/AIDS (UNAIDS), The World Bank Global HIV/AIDS Program (GHAP) (2009). Global AIDS M&E Team (GAMET), Kenya National AIDS Control Council. Kenya HIV prevention response and Modes of Transmission analysis.

[22] Joint United Nations Programme on HIV/AIDS (UNAIDS), Global AIDS M&E Team (GAMET) (2009). Lesotho National AIDS Commission. Lesotho HIV prevention response and modes of transmission analysis.

[23] Joint United Nations Programme on HIV/AIDS (UNAIDS) (2009). The World Bank Global AIDS M&E Team (GAMET), National Emergency Council on HIV and AIDS (NERCHA), Swaziland. Swaziland HIV prevention response and modes of transmission analysis.

[24] Joint United Nations Programme on HIV/AIDS (UNAIDS) (2009). Uganda AIDS Commission. Uganda HIV modes of transmission and prevention response analysis.

[25] Joint United Nations Programme on HIV/AIDS (UNAIDS) (2009). Zambia National HIV/AIDS/STI/TB Council. Zambia: HIV prevention response and Modes of Transmission analysis.

[26] Joint United Nations Programme on HIV/AIDS (UNAIDS) (2010). The World Bank, Zimbabwe National AIDS CouncilM. Zimbabwe: analysis of HIV epidemic, response and Modes of Transmission.

[27] Joint United Nations Programme on HIV/AIDS (UNAIDS) (2012). Data synthesis on tendencies of the HIV epidemic in the Republic of Moldova 2011.

[28] Joint United Nations Programme on HIV/AIDS (UNAIDS) (2010). National Epidemiology Center, Department of Health, Philippines. Short-term estimates of adult HIV incidence in the Philippines in 2010 by mode of transmission.

[29] Joint United Nations Programme on HIV/AIDS (UNAIDS) Consejo Presidencial del SIDA, Dirección General de Infecciones de Transmisión Sexual y SIDA (2010). HIV modes of transmission model: analysis of the distribution of new HIV infections in the Dominican Republic and recommendations for prevention.

[30] Joint United Nations Programme on HIV/AIDS (UNAIDS) USAID, PASCA, Ministerio de Salud (2011). MOT Modelo: Modelo para el análisis de la distribución de nuevas infecciones por el VIH en los grupos de exposición y recomendaciones para la prevención. El Salvador.

[31] Joint United Nations Programme on HIV/AIDS (UNAIDS) (2012). Modes of HIV transmission in Jamaica: distribution of new HIV infections in Jamaica for 2012: recommendations for efficient resource allocation and prevention strategies.

[32] Joint United Nations Programme on HIV/AIDS (UNAIDS) (2012). Comision Nicaraguense del SIDA, Ministerio de Salud, Nicaragua. Modelo de Modos de Transmisión del VIH: Análisis de la distribución de nuevas infecciones por el VIH y recomendaciones para prevención.

[33] Joint United Nations Programme on HIV/AIDS (UNAIDS) (2009). Organización Panamericana de la Salud (OMS). Modos de Transmisión del VIH en América Latina: Resultados de la aplicación del modelo.

[34] Joint United Nations Programme on HIV/AIDS (UNAIDS) (2012). The World Bank. New HIV infections by mode of transmission in West Africa: a multicountry analysis, 2010.

[35] Joint United Nations Programme on HIV/AIDS (UNAIDS) What do we know? Guyana’s modes of transmission study experience.

[36] Vickerman P, Foss AM, Pickles M, Deering K, Verma S, Demers E (2010). To what extent is the HIV epidemic in southern India driven by commercial sex? A modelling analysis. AIDS.

[37] Rojanapithayakorn W (2006). The 100% condom use programme in Asia. Reprod Health Matters.

[38] Joint United Nations Programme on HIV/AIDS (UNAIDS) (2002). Report on the global HIV/AIDS epidemic. http://whqlibdoc.who.int/unaids/2002/global_report_2002.pdf.

[39] World Health Organization (WHO) (2007). Regional Office for South-East Asia. HIV/AIDS in the South-East Asia region.

[40] Ministry of Health and Social Development of the Russian Federation (2008). The Federal Service for Surveillance of Consumer Rights Protection and Human Well-Being of the Russian Federation. Country progress report of the Russian Federation on the implementation of the declaration of commitment on HIV/AIDS.

[41] Joint United Nations Programme on HIV/AIDS (UNAIDS)/World Health Organization (WHO) Working Group on Global HIV/AIDS and STI Surveillance (2004). UNAIDS/WHO epidemiological fact sheets on HIV/AIDS and sexually transmitted infections – Honduras. http://data.unaids.org/publications/fact-sheets01/honduras_en.pdf.

[42] Joint United Nations Programme on HIV/AIDS (UNAIDS) (2013). Spectrum/EPP 2013. http://www.unaids.org/en/dataanalysis/datatools/spectrumepp2013.

[43] Brown T, Peerapatanapokin W (2004). The Asian epidemic model: a process model for exploring HIV policy and programme alternatives in Asia. Sex Transm Infect.

[44] Mishra S, Pickles M, Blanchard JF, Moses S, Boily MC (2014). Distinguishing sources of HIV transmission from the distribution of newly acquired HIV infections: why is it important for HIV prevention planning?. Sex Transm Infect.

[45] Prudden HJ, Watts CH, Vickerman P, Bobrova N, Heise L, Ogungbemi MK (2013). Can the UNAIDS modes of transmission model be improved?: a comparison of the original and revised model projections using data from a setting in west Africa. AIDS.

[46] Delva W, Wilson DP, Abu-Raddad L, Gorgens M, Wilson D, Hallett TB (2012). HIV treatment as prevention: principles of good HIV epidemiology modelling for public health decision-making in all modes of prevention and evaluation. PLoS Med.

[47] Mishra S, Pickles M, Blanchard JF, Moses S, Shubber Z, Boily MC Validation of the modes of transmission model as a tool to prioritize HIV prevention targets: a comparative modeling analysis. PLoS One.

